# Effectiveness of Emergency Care and Life Support Training on Medical Interns' Airway Management Skills: A Pre-Post Study

**DOI:** 10.7759/cureus.113632

**Published:** 2026-07-30

**Authors:** Ganashree C. P., Bharath Sumesh, Geethika R Kandula, Aryan Aryan, Manju Lata

**Affiliations:** 1 Physiology, All India Institute of Medical Sciences, Patna, Patna, IND; 2 Community Medicine, Shimoga Institute of Medical Sciences, Shivamogga, IND; 3 Community Medicine, Ballari Medical College and Research Center, Ballari, IND; 4 Community Medicine, ESIC Medical College and Hospital Bihta, Patna, IND

**Keywords:** emergency airway management, emergency life support training, medical education, pre-post study, simulation based training

## Abstract

Background

Effective airway management is a fundamental competency for medical graduates and is essential for the initial stabilization of critically ill patients. Despite its importance, undergraduate medical training often provides limited structured opportunities for developing and sustaining airway management skills. Emergency Care and Life Support (ECLS) training, integrating simulation-based learning with hands-on practice, may enhance both knowledge retention and clinical competence.

Aim and objective

To evaluate the effectiveness of an ECLS training program in improving airway management knowledge and practical skills among medical interns, and to assess the retention of these competencies following subsequent clinical rotations.

Methods

A prospective pre-post cohort study was conducted over 12 months among 100 medical interns at a tertiary care teaching hospital. Participants underwent a five-day ECLS training program comprising interactive lectures, hands-on skill stations, and simulation-based scenarios focused on initial airway management. Knowledge was assessed using structured scenario-based questionnaires, while practical competence was evaluated using standardized clinical skill checklists. Assessments were performed at four time points: before ECLS training, immediately after training, and following clinical postings in Emergency Medicine, General Medicine, Otorhinolaryngology (ENT), Anesthesiology, and Obstetrics & Gynecology. Changes in scores were analyzed using paired Student’s t-test, with p < 0.05 considered statistically significant.

Results

The mean scenario-based knowledge score increased significantly from 12.56 ± 1.77 before training to 17.74 ± 1.80 immediately after ECLS training (p < 0.001). Baseline clinical skills scores (17.83 ± 0.68) were maintained after the Emergency Medicine rotation (17.73 ± 0.65, p = 0.289) and improved significantly following rotations in General Medicine (18.58 ± 0.50, *p* = 0.001), ENT (18.64 ± 0.48, p = 0.001), and Anesthesiology (19.64 ± 0.48, p = 0.001). In contrast, clinical skills declined significantly after the Obstetrics & Gynecology rotation (17.47 ± 0.50, p = 0.001), suggesting reduced reinforcement of airway management competencies during this posting. Participants demonstrated a sustained increase in self-reported clinical competence and confidence in airway management across successive training milestones, with improvements maintained until the completion of internship.

Conclusion

The ECLS training program significantly enhanced medical interns’ knowledge and practical skills in airway management. Competencies were largely retained or further improved during subsequent clinical rotations with regular exposure to airway management. However, a decline in performance following rotations with limited airway-related clinical experience highlights the need for periodic refresher training and targeted booster sessions to ensure sustained competency throughout internship.

## Introduction

Over the past century, medical education has evolved from a knowledge-based discipline to one that prioritizes competency, patient safety, and measurable clinical performance. This transformation has been driven by increasing healthcare complexity and growing recognition that medical errors remain a major cause of preventable morbidity and mortality. A landmark event in this evolution was the publication of the Institute of Medicine’s report *To Err Is Human *in 1999, which estimated that nearly 98,000 patients die each year in hospitals because of preventable medical errors [1]. The report reinforced the need for educational strategies that ensure healthcare professionals are equipped with the knowledge, skills, and attitudes necessary to deliver safe and effective patient care.

The foundations of modern medical education can be traced to the Flexner Report of 1910, which advocated scientific rigor, standardized curricula, and evidence-based medical training [2]. Although this model profoundly influenced medical education, traditional learning largely relied on apprenticeship, often summarized as “see one, do one, teach one.” Over time, this approach has gradually been replaced by Competency-Based Medical Education (CBME), which emphasizes clearly defined learning outcomes, objective assessment, and demonstration of clinical competence rather than completion of training alone. Educational tools such as the Objective Structured Clinical Examination (OSCE) and the Objective Structured Assessment of Technical Skills (OSATS) have become integral to this transition by enabling standardized evaluation of learners’ cognitive, psychomotor, and communication skills [3].

The principles of CBME are reflected in international competency frameworks such as those developed by the Accreditation Council for Graduate Medical Education (ACGME), which outlines six core competencies expected of every graduating physician [4]. Among these, medical knowledge extends beyond factual understanding and requires learners to integrate biomedical, clinical, epidemiological, and behavioral sciences into safe and effective patient care. Consequently, modern medical curricula increasingly focus on developing competent practitioners who can apply knowledge confidently in real clinical settings.

Despite advances in medical education, healthcare systems continue to face substantial workforce challenges. The *World Health Report 2006, Working Together for Health*, estimated a global shortage of approximately 2.4 million doctors, nurses, and midwives across 57 countries experiencing critical workforce deficits [5]. This shortage highlights the importance of producing not only a sufficient number of healthcare professionals but also graduates who are competent to manage increasingly complex clinical situations from the beginning of their professional careers.

Medical emergencies represent situations in which prompt recognition and timely intervention are critical for reducing mortality and preventing long-term complications. Early assessment and immediate management have a direct influence on patient survival, making emergency care an essential competency for all physicians [6,7]. Among the various approaches to emergency assessment, the Airway, Breathing, Circulation, Disability, and Exposure (ABCDE) framework provides a systematic method for the rapid evaluation and stabilization of critically ill or injured patients. Its simplicity and applicability across both prehospital and hospital settings have made it a universally accepted approach to emergency management [8].

In South Asian countries, particularly within resource-constrained tertiary care hospitals and primary healthcare settings, newly graduated doctors and medical interns are frequently the first responders responsible for the initial assessment and stabilization of patients presenting with life-threatening conditions [9]. Although the Airway, Breathing, Circulation, Disability, and Exposure (ABCDE) approach provides a systematic framework for early assessment, effective management often requires timely airway interventions and cardiopulmonary resuscitation. Consequently, competency in airway management and Emergency Care and Life Support (ECLS) is a fundamental expectation of every medical graduate and is essential for improving emergency preparedness and patient safety in the region.

Historically, these competencies were acquired through direct patient care during apprenticeship-based clinical training. However, contemporary healthcare delivery has reduced opportunities for experiential learning because of shorter hospital stays, limited exposure to critically ill patients, restrictions on resident working hours, and increasing clinical workloads that constrain faculty teaching time. These challenges have prompted medical educators to adopt educational strategies that allow learners to develop procedural skills and clinical decision-making in a structured and risk-free environment before caring for real patients.

Simulation-based education has emerged as one of the most effective responses to these challenges. Rather than replacing clinical experience, simulation enhances it by recreating authentic clinical situations in which learners can repeatedly practice procedures, refine decision-making, improve communication, and receive immediate feedback without jeopardizing patient safety [10,11]. Advances in educational technology have expanded simulation modalities to include task trainers, standardized patients, high-fidelity mannequins, virtual reality platforms, and hybrid simulation models, all of which are now widely incorporated into undergraduate and postgraduate medical curricula [12-14]. Evidence from medical education research consistently demonstrates that simulation-based training improves procedural competence, clinical reasoning, teamwork, learner confidence, and ultimately contributes to safer patient care.

Emergency Cardiac Life Support (ECLS) training integrates these educational principles by combining interactive teaching with simulation-based practice to develop competence in the recognition and management of life-threatening emergencies, particularly airway management and cardiopulmonary resuscitation [15]. Evaluating the effectiveness of such training is essential to determine whether improvements achieved during simulation translate into enhanced knowledge, greater confidence, and improved clinical competency among medical trainees.

Against this background, the present pre-post interventional study was designed to evaluate the impact of a structured Emergency Cardiac Life Support (ECLS) training programme on the knowledge, self-confidence, and clinical competency of medical interns at a tertiary care teaching hospital, with particular emphasis on the acquisition of initial airway management skills required during medical emergencies. Therefore, the primary objective of this study was to determine the impact of the ECLS training programme on medical interns’ knowledge and practical competency in initial airway management. The secondary objectives were to evaluate changes in self-confidence and to assess the retention of airway management competence after subsequent clinical postings.

## Materials and methods

Study design and setting

A pre-post cohort study was conducted over a period of 12 months from 01/09/2022 to 05/06/2023 at the Medical Education Unit of a tertiary care teaching hospital. The study was approved by the Institutional Ethics Committee of Basaveshwera Medical College and Hospital (Approval No. BMC&H/IEC/111/2022-2023 dated 28/07/2022). Written informed consent was obtained from all participants prior to enrolment.

Sample size

This was a prospective educational cohort study involving all eligible medical interns undergoing compulsory internship during the 12-month study period at the institution. Therefore, a consecutive sampling approach was adopted, and all 100 eligible interns were included. The sample size was determined by the number of interns available during the study period rather than by an *a priori* statistical calculation. This census-based approach minimized selection bias and ensured adequate representation across all clinical rotations.

Study participants

The study population comprised medical interns undergoing compulsory rotatory internship at the host institution during the study period. A universal sampling strategy was adopted, whereby all eligible interns were invited to participate, ensuring comprehensive representation of the internship cohort. Eligible participants were required to provide written informed consent and attend the complete five-day Emergency Care and Life Support (ECLS) training programme. Interns who declined participation or were absent from any component of the training programme or assessment were excluded from the study.

Intervention

The intervention consisted of a structured five-day Emergency Care and Life Support (ECLS) training programme conducted in the institutional Medical Skills Laboratory. The programme was delivered by certified ECLS instructors from Jeeva Raksha Trust, affiliated with Rajiv Gandhi University of Health Sciences, Bengaluru. The curriculum integrated interactive didactic lectures, hands-on skill stations, and high-fidelity simulation-based scenarios to provide comprehensive training in the recognition and management of medical emergencies.

Particular emphasis was placed on the systematic assessment and initial management of airway emergencies using the Airway, Breathing, Circulation, Disability, and Exposure (ABCDE) approach. Practical sessions included airway assessment, bag-mask ventilation, insertion and use of basic airway adjuncts, oxygen delivery techniques, cardiopulmonary resuscitation, and team-based emergency response. At the end of the programme, all participants underwent a structured practical assessment of airway management skills conducted by certified ECLS trainers using standardized competency criteria.

Assessment of outcomes

Participants were assessed at four predefined time points to evaluate knowledge acquisition, clinical competency, and skill retention. Baseline knowledge, clinical reasoning, and decision-making abilities were assessed before commencement of the training using a structured scenario-based questionnaire. Immediately after completion of the ECLS programme, participants completed the same scenario-based assessment to determine the immediate educational impact of the intervention.

Practical competency in airway management was evaluated at the end of the training programme using a standardized clinical skills checklist administered by certified ECLS trainers. To determine long-term retention and transfer of learning into clinical practice, participants were reassessed after 16 weeks of completion of their departmental postings in Emergency Medicine, General Medicine, Obstetrics and Gynaecology, Otorhinolaryngology (ENT), and Anaesthesiology using the same standardized competency checklist.

Study instruments

Knowledge, clinical reasoning, and decision-making were assessed using an investigator-developed scenario-based questionnaire specifically designed for this study. Clinical competency in airway management was evaluated using a standardized airway management skills checklist. Both instruments were developed in accordance with contemporary emergency care guidelines and the predefined learning objectives of the ECLS curriculum.

The content validity of the questionnaire and checklist was established through review by a panel of certified ECLS instructors with expertise in emergency care education. The instruments were refined based on expert feedback to ensure relevance, clarity, and alignment with the intended learning outcomes. As these are original investigator-developed tools, the complete questionnaire and clinical skills checklist are provided as supplementary appendices to this manuscript.

Data collection

Data were collected using both electronic and paper-based methods. Participants completed the knowledge assessments through Google Forms, while practical competency assessments were recorded on standardized paper-based evaluation forms by certified instructors. All completed questionnaires and assessment checklists were reviewed for completeness and consistency before data entry. The data were subsequently entered into Microsoft Excel (Microsoft Corporation, Redmond, USA) and independently cross-verified to minimize transcription errors prior to statistical analysis.

Outcome measures

The primary outcome of the study was the improvement in medical interns’ knowledge and competency in initial airway management following completion of the ECLS training programme. Knowledge acquisition was measured using scenario-based assessment scores, whereas practical competency was evaluated using standardized clinical skills assessment checklists.

Secondary outcomes included changes in participants’ self-confidence in managing emergency airway situations, immediate post-training clinical competency scores, and the retention of airway management skills following completion of relevant clinical postings. These outcomes were assessed to determine the sustainability of learning and the influence of subsequent clinical exposure on maintaining airway management competence.

Statistical analysis

Data were analysed using SPSS® version 26.0 (IBM Corp., Armonk, NY, USA) for Windows. Continuous variables were expressed as mean ± standard deviation (SD), while categorical variables were summarized as frequencies and percentages. Comparisons between pre- and post-training knowledge scores and clinical competency scores were performed using the paired Student’s t-test. Comparisons of competency scores following departmental postings were also analysed using paired t-tests where appropriate. A two-tailed p-value <0.05 was considered statistically significant.

## Results

Study population

A total of 100 medical interns participated in the study. The mean age of the participants was 23.32 ± 0.49 years, and 59 (59%) were female.

Scenario-based knowledge assessment

Table 1 compares the participants’ baseline knowledge and clinical skills scores with subsequent assessments across different training milestones. The findings demonstrate significant improvements in theoretical knowledge and practical clinical skills following Emergency Care and Life Support (ECLS) training, with sustained gains observed after departmental postings.

**Table 1:** Comparison of ECLS Knowledge and Clinical Skills Scores Across Training Milestones Relative to Baseline Knowledge scores Data are presented as mean ± standard deviation (SD). Baseline scores were obtained before Emergency Cardiovascular Life Support (ECLS) training. Follow-up assessments were conducted at the end of the ECLS scenario-based training, after the ECLS clinical skills assessment, and at the end of internship. Mean differences represent the change from baseline. Statistical comparisons were performed using paired t-tests. A p-value < 0.05 was considered statistically significant. *** indicates p < 0.001.

Evaluation Milestone	Baseline Mean ± SD	Mean Score ± SD	Mean Difference	t-statistic	p-value	Significance
End of ECLS Scenario Survey	1.96 ± 0.82	7.78 ± 0.82	+5.82	53.59	< 0.0001	Extremely Significant (***)
End of ECLS Clinical Skills training	1.96 ± 0.82	8.05 ± 0.85	+6.09	49.44	< 0.0001	Extremely Significant (***)
End of Internship	1.96 ± 0.82	8.35 ± 0.63	+6.39	61.82	<0.0001	Extremely Significant (***)

The mean scenario-based assessment score increased significantly following the Emergency Care and Life Support (ECLS) training programme. The mean pre-training score improved from 12.56 ± 1.77 to 17.74 ± 1.80 immediately after training, representing a statistically significant improvement (p < 0.001).

Clinical skills assessment

Table 2 presents the comparison of clinical skills assessment scores immediately after ECLS training and following departmental postings. The findings highlight the retention and progression of clinical competencies over time.

**Table 2:** Comparison of Clinical Skills Assessment Scores Immediately After Emergency Care and Life Support (ECLS) Training and Following Departmental Postings Among Medical Interns (n = 100) Values are presented as mean ± standard deviation (SD) with 95% confidence intervals (CI). The immediate post-ECLS clinical skills assessment served as the baseline for comparison. ^†^P-values were calculated using the paired Student’s t-test to compare clinical skills scores after each departmental posting with the immediate post-ECLS assessment. A two-tailed p-value < 0.05 was considered statistically significant. *Statistically significant difference (p < 0.05). ECLS = Emergency Care and Life Support; ENT = Otorhinolaryngology; CI = Confidence Interval; SD = Standard Deviation.

Department	Mean ± SD	95% CI	P-value^†^
Post-ECLS (baseline)	17.83 ± 0.68	17.70-17.96	-
Emergency Medicine	17.73 ± 0.65	17.60-17.86	0.289
General Medicine	18.58 ± 0.496	18.48-18.68	0.001*
Obstetrics & Gynecology	17.47 ± 0.502	17.37-17.57	0.001*
ENT	18.64 ± 0.48	18.54-18.74	0.001*
Anesthesia	19.64 ± 0.482	19.54-19.74	0.001*

The mean clinical skills score immediately after ECLS training was 17.83 ± 0.68. Scores remained comparable following the Emergency Medicine posting (17.73 ± 0.65), with no statistically significant difference from the immediate post-training assessment (p = 0.289).

Significant improvements in clinical skills were observed after the General Medicine (18.58 ± 0.50), ENT (18.64 ± 0.48), and Anaesthesiology (19.64 ± 0.48) postings compared with the immediate post-ECLS assessment (all p = 0.001). In contrast, the mean clinical skills score decreased significantly following the Obstetrics and Gynaecology posting (17.47 ± 0.50, p = 0.001).

Among all departmental postings, the highest clinical skills score was recorded after the Anaesthesiology posting, whereas the lowest score was observed following the Obstetrics and Gynaecology posting.

Table 3 summarizes the longitudinal changes in self-reported clinical competence and airway management confidence across the three assessment milestones. The results demonstrate progressive improvement in confidence and perceived competence following ECLS training and subsequent clinical exposure.

**Table 3:** Longitudinal Cohort Analysis of Self-Reported Clinical Competence and Airway Skill Confidence Across Training Milestones (N = 100) Values are presented as mean ± standard deviation (SD). Self-reported clinical competence and airway skill confidence were measured on a 10-point interval scale (1 = lowest confidence; 10 = highest confidence). Comparisons between baseline and each subsequent assessment were performed using a paired t-test. Degrees of freedom (df) = 99 for all comparisons. *** indicates p < 0.001.

Evaluation Milestone	Mean Score ± SD	Baseline Mean ± SD	Mean Difference	t-value	p-value	Statistical Significance
End of ECLS Scenario Survey	7.78 ± 0.82	1.96 ± 0.82	+5.82	53.59	<0.0001	Extremely Significant (***)
End of ECLS Clinical Skills	8.05 ± 0.85	1.96 ± 0.82	+6.09	49.44	<0.0001	Extremely Significant (***)
End of Internship	8.35 ± 0.63	1.96 ± 0.82	+6.39	61.82	<0.0001	Extremely Significant (***)

## Discussion

In medical education, competency-based training has become the foundation of modern curricula, shifting the focus from knowledge acquisition alone to the development of measurable clinical competencies [16]. Within this framework, simulation-based education has emerged as an essential teaching strategy, providing learners with a safe and structured environment to acquire, practice, and refine clinical skills before applying them in real patient care. By recreating realistic clinical scenarios, simulation enables repeated practice, immediate feedback, and reflective learning, thereby improving technical skills, clinical decision-making, communication, and confidence while maintaining patient safety. Consequently, simulation has become an integral component of undergraduate and postgraduate medical training worldwide. Gaba described simulation as a powerful educational strategy that allows trainees to learn from mistakes without compromising patient safety, while Lateef emphasized its role in bridging the gap between classroom learning and real-world clinical practice.

In the present study, participation in the Emergency Care and Life Support (ECLS) programme significantly improved medical interns’ knowledge, as reflected in higher scenario-based assessment scores immediately after training. These findings suggest that structured simulation-based teaching is an effective method for reinforcing core concepts in emergency care and improving learners’ preparedness to manage critically ill patients.

Our findings are in line with those reported by Wayne et al., who demonstrated that simulation-based Advanced Cardiac Life Support (ACLS) training significantly improved the resuscitation performance of internal medicine residents [17]. Residents who received structured simulator-based training outperformed those who relied solely on routine clinical experience, highlighting that workplace exposure alone may not be sufficient for developing critical emergency skills. Similarly, the substantial improvement observed among our interns indicates that simulation-based ECLS training provides a strong educational foundation for developing competencies in emergency airway management and resuscitation.

The educational value of simulation has also been highlighted by Mancera et al. [13], who found that emergency medical service personnel across different levels of training regarded simulation as an engaging and effective learning method. Participants expressed strong support for incorporating simulation into continuing professional education. The positive changes observed in our study, including improvements in both knowledge and practical airway management skills, further reinforce the role of simulation as an effective teaching strategy that should be integrated into undergraduate medical education.

Beyond improving theoretical knowledge, the ECLS programme produced a marked enhancement in clinical airway management skills immediately after training. This finding supports the growing body of evidence that hands-on simulation helps learners translate theoretical concepts into practical competence. Rosenthal et al. similarly reported that simulation offers a safe and reliable platform for teaching and assessing emergency airway management skills, although they also noted that the type and fidelity of the simulator may influence learning outcomes [18].

The effectiveness of simulation-based training has been consistently demonstrated in the literature. In a systematic review and meta-analysis, Cook et al. concluded that technology-enhanced simulation leads to substantial improvements in knowledge, procedural skills, and professional behaviours compared with traditional teaching alone. Importantly, they also reported moderate improvements in patient-related outcomes, highlighting the potential of simulation to improve the quality and safety of healthcare delivery [19].

One of the most encouraging findings of the present study was the continued improvement in airway management skills during internship postings in Emergency Medicine, General Medicine, ENT, and Anaesthesiology. This suggests that simulation serves as an effective foundation on which clinical experience can be built. As interns encountered real patients and repeatedly performed airway-related procedures under supervision, their competency continued to improve. Similar observations have been reported by McGaghie et al., who demonstrated that skills acquired through simulation are better retained when reinforced by repeated clinical practice [20]. The highest competency scores following the Anaesthesiology rotation were expected, given the frequent exposure to airway assessment and airway management procedures in that specialty.

In contrast, interns demonstrated a decline in competency after completing their Obstetrics and Gynaecology posting. This likely reflects the limited opportunities to perform airway management during that rotation, resulting in reduced reinforcement of previously acquired skills. Previous research has consistently shown that procedural skills deteriorate when they are not practiced regularly. This finding highlights the importance of periodic refresher training and ongoing simulation sessions to maintain competency throughout the internship [21].

The findings of the present study demonstrate that the ECLS training programme was effective in improving medical interns’ knowledge, practical competency, and self-confidence in initial airway management. While simulation-based training constituted a key instructional component of the programme, the observed improvements should be interpreted as the cumulative effect of the comprehensive ECLS curriculum, which integrated didactic teaching, structured demonstrations, hands-on skill stations, simulation scenarios, and supervised feedback. Simulation facilitated experiential learning and deliberate practice; however, it was the multimodal design of the training programme that likely contributed to the significant gains in competence and confidence observed among participants. These findings support the effectiveness of structured ECLS training as a comprehensive educational intervention for preparing medical interns to manage airway emergencies.

Overall, our findings indicate that structured ECLS training is an effective educational intervention for improving both knowledge and practical skills in emergency airway management among medical interns. The variation in performance across different clinical postings also underscores the importance of continuous practice and reinforcement. Integrating simulation-based refresher sessions throughout the internship may help sustain competency and ensure that newly qualified doctors remain prepared to respond confidently and effectively to medical emergencies.

This study has several limitations that should be considered while interpreting the findings. The absence of a control group limits the ability to attribute the observed improvements solely to the ECLS training programme. The pre- and post-test design may have introduced a testing effect, and assessor bias cannot be completely excluded despite the use of standardized rubric-based assessments by certified ECLS instructors. Although the assessment tools were based on established ECLS competency criteria, formal psychometric validation was not undertaken. Furthermore, this was a single-centre study conducted at a tertiary care teaching institution using a pre-post study design, which may limit the generalizability of the findings. Competency was assessed using simulation-based evaluations and supervised clinical assessments during internship rather than direct observation of routine clinical practice, and long-term retention of knowledge and skills was not evaluated. Future multicentre studies incorporating control groups, validated assessment instruments, larger sample sizes, and longer follow-up are needed to determine whether the improvements observed after ECLS training are sustained over time and translate into improved patient care and clinical outcomes.

## Conclusions

The findings of this study suggest that participation in the Emergency Care and Life Support (ECLS) training programme was associated with significant improvements in medical interns’ knowledge, practical competency, and retention of airway management skills. Although competency was reinforced during most clinical postings, targeted reinforcement strategies may be beneficial during rotations with limited airway management exposure. These findings support the integration of structured ECLS training, along with periodic refresher sessions and simulation-based reinforcement, into internship programmes to promote sustained competency. In the context of South Asian healthcare systems, where newly graduated doctors frequently provide emergency care in resource-constrained primary and secondary healthcare settings, such training programmes have the potential to strengthen emergency preparedness and contribute to improved patient safety.

## References

[1] Institute of Medicine (US) Committee on Quality of Health Care in America (2000). To Err is Human: Building a Safer Health System. https://pubmed.ncbi.nlm.nih.gov/25077248/.

[2] Yoon HB, Myung SJ (2024). The impact and implications of the Flexner Report on medical education in Korea. J Korean Med Sci.

[3] Shahzad A, Saeed MH, Paiker S (2017). Dental students' concerns regarding OSPE and OSCE: a qualitative feedback for process improvement. BDJ Open.

[4] Stanford Medicine (2026). ACGME core competencies | Graduate Medical Education | Stanford Medicine. http://2026.

[5] World Health Organization (2006). The World Health Report : 2006 : Working Together for Health. https://iris.who.int/handle/10665/43432.

[6] Ramanayake RP, Ranasingha S, Lakmini S (2014). Management of emergencies in general practice: role of general practitioners. J Family Med Prim Care.

[7] Johnston CL, Coulthard MG, Schluter PJ, Dick ML (2001). Medical emergencies in general practice in south-east Queensland: prevalence and practice preparedness. Med J Aust.

[8] Thim T, Krarup NH, Grove EL, Rohde CV, Løfgren B (2012). Initial assessment and treatment with the Airway, Breathing, Circulation, Disability, Exposure (ABCDE) approach. Int J Gen Med.

[9] Suryawanshi DM, Surekha A, Divya R, Gunasekaran K, Malini I (2022). Awareness and preparedness of first responders regarding chemical, biological, radiological, nuclear and explosive (CBRNE) disaster management of a tertiary medical institute in South India: A mixed methods study. J Family Med Prim Care.

[10] Lateef F (2010). Simulation-based learning: Just like the real thing. J Emerg Trauma Shock.

[11] Gaba D (1999). Human work environment and simulators. Anaesthesia.

[12] Gaba DM (2004). The future vision of simulation in health care. Qual Saf Health Care.

[13] Mancera M, Genthe N, Gussick M, Lohmeier M, Thompson R, Shah M (2022). Acceptability and preferences of simulation-based continuing education among emergency medical service providers. WMJ.

[14] Elendu C, Amaechi DC, Okatta AU, Amaechi EC, Elendu TC, Ezeh CP, Elendu ID (2024). The impact of simulation-based training in medical education: A review. Medicine (Baltimore).

[15] (2026). Saving lives through emergency care training | JeevaRaksha. https://jeevaraksha.org/.

[16] Kurien M, Thomas K (2024). Competency Based Medical Education (CBME) in India: clinicians' multifaceted challenges. Indian J Otolaryngol Head Neck Surg.

[17] Wayne DB, Butter J, Siddall VJ (2005). Simulation-based training of internal medicine residents in advanced cardiac life support protocols: a randomized trial. Teach Learn Med.

[18] Rosenthal E, Owen H (2004). An assessment of small simulators used to teach basic airway management. Anaesth Intensive Care.

[19] Cook DA, Hatala R, Brydges R (2011). Technology-enhanced simulation for health professions education: a systematic review and meta-analysis. JAMA.

[20] McGaghie WC, Issenberg SB, Barsuk JH, Wayne DB (2014). A critical review of simulation-based mastery learning with translational outcomes. Med Educ.

[21] Tatel CE, Ackerman PL (2025). Procedural skill retention and decay: a meta-analytic review. Psychol Bull.

